# Autoregulation of *greA* Expression Relies on GraL Rather than on *greA* Promoter Region

**DOI:** 10.3390/ijms20205224

**Published:** 2019-10-22

**Authors:** Maciej Dylewski, Llorenç Fernández-Coll, Bożena Bruhn-Olszewska, Carlos Balsalobre, Katarzyna Potrykus

**Affiliations:** 1 Department of Bacterial Molecular Genetics, Faculty of Biology, University of Gdańsk, W. Stwosza 59, 80-299 Gdańsk, Poland; maciej.dylewski@phdstud.ug.edu.pl (M.D.); bruhn22@gmail.com (B.B.-O.); 2 Department of Genetics, Microbiology and Statistics, Faculty of Biology, University of Barcelona, Av. Diagonal 643, 08028 Barcelona, Spain; llorenc.fernandezcoll@nih.gov (L.F.-C.); cbalsalobre@ub.edu (C.B.)

**Keywords:** *greA*, GraL, autoregulation, sRNA, RNAP, transcription regulation, gene expression, intrinsic terminator

## Abstract

GreA is a well-characterized transcriptional factor that acts primarily by rescuing stalled RNA polymerase complexes, but has also been shown to be the major transcriptional fidelity and proofreading factor, while it inhibits DNA break repair. Regulation of *greA* gene expression itself is still not well understood. So far, it has been shown that its expression is driven by two overlapping promoters and that *greA* leader encodes a small RNA (GraL) that is acting *in trans* on *nudE* mRNA. It has been also shown that GreA autoinhibits its own expression *in vivo*. Here, we decided to investigate the inner workings of this autoregulatory loop. Transcriptional fusions with *lacZ* reporter carrying different modifications (made both to the *greA* promoter and leader regions) were made to pinpoint the sequences responsible for this autoregulation, while GraL levels were also monitored. Our data indicate that GreA mediated regulation of its own gene expression is dependent on GraL acting *in cis* (a rare example of dual-action sRNA), rather than on the promoter region. However, a yet unidentified, additional factor seems to participate in this regulation as well. Overall, the GreA/GraL regulatory loop seems to have unique but hard to classify properties.

## 1. Introduction

Gene expression regulation can take place at several levels, e.g., at the transcriptional, post-transcriptional, and translational level. In bacteria, transcriptional regulation usually occurs at the stage of initiation, i.e., activators or repressors bind to a specific promoter region sequence, and enhance or inhibit transcription, respectively. Transcription could also be regulated at the stage of elongation (e.g., by the *Escherichia coli* GreA and GreB proteins that directly interact with RNA polymerase (RNAP), see below), or at the stage of termination (e.g., by antitermination factors, such as the Nus factors (NusA, NusB, NusG and NusE), which form a complex interaction with both RNAP and specific RNA sequences [1]). Other examples include attenuation mediated by ribosomes or regulation by RNA aptamers bound to RNAP [2,3,4].

A large group of transcriptional regulators also includes small RNA (sRNA). These short RNA species, ranging in size from 50 to 500 nucleotides (nt) [5], were shown to either act *in cis* (e.g., antisense RNAs [5] and riboswitches [3]) or *in trans*, where they enhance or inhibit translation of their targets by diverse mechanisms, relying on complementary base-pairing [6]. *Trans* acting sRNAs often require a protein chaperone mediating sRNA-target mRNA interaction, called Hfq [5].

GreA transcription factor is responsible for rescuing stalled RNAP–DNA complexes that arise as a result of pausing or backtracking, i.e., when polymerase translocates backwards and the 3′ end of the nascent RNA transcript is moved out of register [7,8]. Its structure is L-shaped, with the globular C-terminal domain interacting with the β’ subunit rim domains at the entrance to the RNAP secondary channel, and the *N*-terminal finger-like domain being inserted into the secondary channel itself [9]. It is worth mentioning that it is generally accepted that NTP (nucleotide triphosphate) substrates are delivered to the RNAP catalytic center through that channel [10,11]. At the very tip of GreA’s finger domain are two conserved acidic residues (D41 and E44), necessary to induce the intrinsic endonucleolytic activity of RNAP and thus rescue the stalled complex [9]. It is thought that GreA is not bound to RNAP all the time; in addition, other secondary channel proteins exist, e.g., GreB (a very close GreA homologue), DksA, TraR, or Gfh1 [12,13,14,15]. It was shown that there is competition between GreA, GreB, and DksA for binding to RNAP [16,17,18], as well as that different modifications induced by environmental changes may affect a given secondary channel protein’s ability to bind to RNAP (reviewed in [19]).

In addition, GreA was also demonstrated to act at the transcription initiation level, e.g., by enhancing promoter escape [20] or by affecting the RNAP–DNA complex isomerization at the ribosomal *rrnB* P1 promoter [16]. Interestingly, GreA was recently identified as the major transcriptional proofreading and transcription fidelity factor in *Escherichia coli* [21,22]. Furthermore, GreA/GreB homologues are widely conserved in other bacteria, and were shown to be necessary for expression of genes related to host invasion (e.g., *Salmonella enteritica* [23]), and in one case reported to be essential genes in *Mycoplasma pneumoniae* [24].

**Figure 1:**
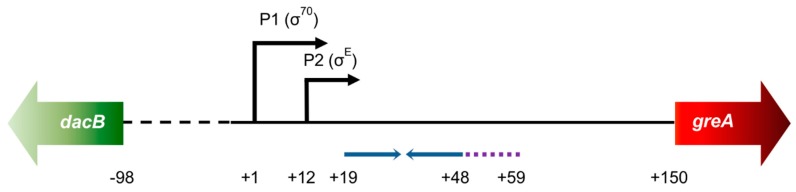
Schematic representation of the *greA* promoter and leader region. Black arrows indicate P1 (σ^70^-dependent) and P2 (σ^E^-dependent) promoters. Blue arrows indicate complementary sequences that form the GraL terminator hairpin. Purple dotted line—U-rich sequence where GraL is imprecisely terminated. The first AUG codon of *greA* is at position +150; the first AUG codon of *dacB* is at position −98. All numbering is in respect to the P1 transcription start site. Not drawn to scale.

Although much is known about GreA’s action, especially in *E. coli*, regulation of its expression is still not fully understood. In our previous work [25], we mapped *greA* promoters and showed that GreA protein autoinhibits transcription of its own gene *in vivo*, which was evidenced by monitoring β-galactosidase activities of the p*greA* promoter region—*lacZ* fusion in either Δ*greA* background or when GreA was overproduced from a plasmid. In these systems, transcriptional activation (derepression) and transcriptional inhibition were observed, respectively. Still, such a phenomenon was not observed under *in vitro* transcription conditions in a fully defined system where increasing GreA concentrations were employed. In addition, that study yielded the discovery of GraL, a small RNA encoded in the *greA* leader region. We had shown that only about 1/3 of transcripts whose transcription is initiated at the two *greA* promoters (P1 and P2) is able to proceed through the intrinsic terminator embedded in the *greA*’s 5′ untranslated region (UTR) and form full-length *greA* mRNA [25]. Interestingly, that transcription termination is highly imprecise, yielding two sets of transcripts: ranging from 49 to 59 nt (transcripts originated from the P1 promoter) and from 37 to 47 nt (P2 promoter originated). The short transcripts were called GraL, and we have recently shown that GraL displays effects as an in trans acting sRNA [26]. A schematic representation of the *greA* promoter and leader region is presented in Figure 1.

Here, we decided to dissect the mechanism of GreA mediated inhibition of *greA* gene expression *in vivo* by investigating at which stage of transcription this regulation takes place and whether GraL plays any *in cis* role in this process. We found that GreA autoregulation is promoter region independent, while it relies on the GraL sequence, both upstream of the terminator and forming the terminator hairpin. Thus, GraL is not only an unusual example of imprecise termination but it also seems to represent a new paradigm of an sRNA acting both *in cis* and *in trans*. Moreover, feedback inhibition by GreA *in vivo* is nearly completely abolished by a mutation that eliminates the ability of GreA to cleave backtracked nascent RNA. Although many questions still remain, our data furthers the understanding of how gene expression of the GreA global transcriptional regulator is controlled.

## 2. Results

### 2.1. GreA Autoregulation Depends on Catalytically Active GreA

In the first step of our investigation, we wanted to explore whether the previously observed inhibition of *greA* expression by GreA depends on the GreA’s antipausing activity and ability to induce the intrinsic endonucleolytic activity of RNAP. For this purpose, we used a GreA D41A mutant that has been shown to completely lose its antipause effect on RNAP activity but that is still able to bind to the RNAP secondary channel [12]. A region spanning from −1030 to +175 of *greA* (where +1 corresponds to the transcription initiation site of the *greA* P1 promoter) was fused to the *lacZ* reporter gene and introduced onto the chromosome in a single copy. β-galactosidase activity was monitored in wild-type (Δ*lacZ*) and corresponding Δ*greA* cells, bearing either a vector control (pBR322), a plasmid encoding wild-type (wt) GreA (pBR–GreA), or its catalytically inactive counterpart (pBR–GreAD41A), cloned under its native promoters.

**Figure 2:**
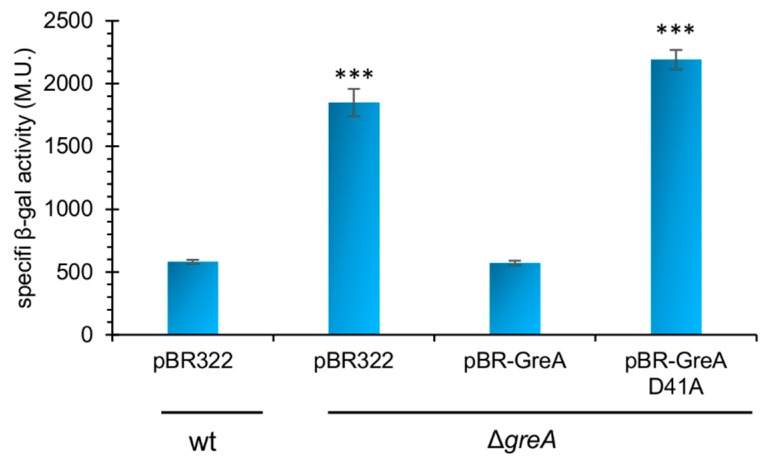
Catalytically active GreA is required for GreA autoregulation. Transcrip tional activity of the *greA* promoter and leader region (−1030 to +175) was measured by monitoring β-galactosiadase activity of the p*greA–lacZ* fusion in different strain backgrounds: wt—wild type (LFC3–MG1655 Δ*lacZ* [17]), Δ*greA* (LFC4–MG1655 Δ*lacZ* Δ*greA*); pBR-322—vector control; pBR–GreA—vector bearing *greA* gene under its native promoter region (i.e., GraL was present as well); pBR–GreAD41— the same as pBR-GreA but encoding catalytically inactive GreA. Cells were grown at 37 °C in LB (Luria Broth) with aeration; at OD_600_ ~ 1.5 samples were removed and β-galactosidase activity was measured. Average results from three independent experiments are shown. Error bars represent S.D. For clarity, *p*-values calculated for wt pBR322 *vs*. Δ*greA* strains are only shown. *** *p* < 0.0005.

As shown in Figure 2 and reported previously [25], in the absence of GreA, transcriptional activity of the p*greA*–*lacZ* fusion is significantly increased (about 3.5-fold). Reintroduction of native GreA complemented the Δ*greA* strain in respect to bringing back the fusion’s β-galactosidase activity to the wt levels. Importantly, under the same conditions, the GreA D41A mutant was unable to repress *greA* expression, implying not only that the mechanism of GreA autoregulation involves transcription but also that this mechanism is likely to require cleavage of backtracked RNA. This also suggests that GreA autoregulation might take place at a stage other than transcriptional initiation since a double GreA mutant [D41A E44A] was previously shown to retain its regulation at this step of transcription in case of another promoter *in vivo* [18].

### 2.2. GreA Autoregulation is Independent of the Promoter Region

**Figure 3:**
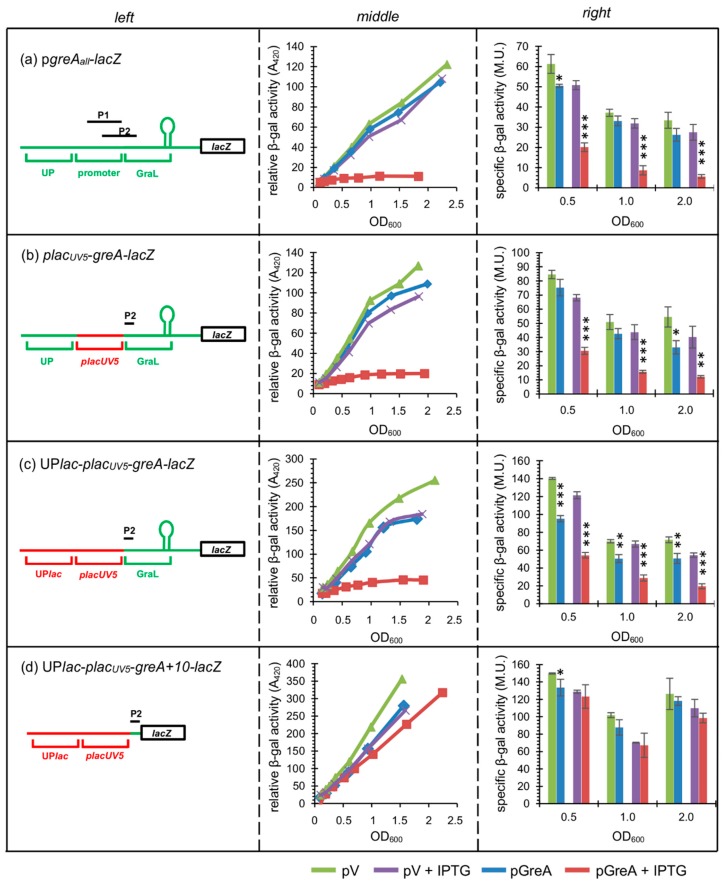
The promoter region does not determine autoregulation exerted by GreA. Assessment of β-galactosidase activity in strains overproducing GreA from a multicopy plasmid (pGreA) for: (**a**) p*greA*_all_-*lacZ;* (**b**) p*lac*_UV5_-*greA*-*lacZ;* (**c**) UP-p*lac*_UV5_-*greA*-*lacZ*; (**d**) UP-p*lac*_UV5_-*greA*+*10*-*lacZ* (negative control) fusions. Wt Δ*lacZ* strains carrying appropriate single copy fusions were grown in LB at 32 °C with aeration; if present, isopropyl-β-d-thio-galactopyranoside (IPTG) was added at OD_600_ ~ 0.1 to 1 mM; pV—vector control. Left panels—schematic representation of the *lacZ* fusions employed; hairpin symbolizes the GraL terminator; not drawn to scale. Middle panels—differential plots of β-galactosidase activities *vs*. OD_600_, following transcriptional activity throughout growth. The slope of each curve corresponds to the specific activity of β-galactosidase. Right panels—specific β-galactosidase activities assessed for each strain at OD_600_ ~ 0.5, 1.0, and 2.0 (experiment was done in triplicate). Error bars represent S.D. For clarity, *p*-values calculated for pV *vs*. pGreA, and pV+IPTG *vs*. pGreA+IPTG are only shown. * *p* < 0.05, ** *p* < 0.005, *** *p* < 0.0005.

To verify in another way that GreA autoregulation is independent of the transcription initiation step, several single-copy transcriptional fusions with the *lacZ* reporter gene were constructed, methodically changing the promoter region elements (Figure 3, left panels). The basic fusion contained a *greA* region spanning −100 to +136 bp, where +1 again corresponds to the P1 promoter transcription initiation site (p*greA*_all_-*lacZ*). The *greA* promoter region (−1 to −36) was then replaced by the p*lac*_UV5_ promoter, while other elements were retained (p*lac*_UV5_-*greA*-*lacZ* fusion). In another construct, the region upstream of the promoter was replaced by the p*lac* UP region in addition to the promoter replacement (UP-p*lac*_UV5_-*greA*-*lacZ* fusion). In addition, the CRP (catabolite repressor protein) binding site was mutated so as to abolish any possible effects of catabolite repression that regulates p*lac* expression. The changes were at positions −66 (G->A) and −55 (C->T) [27]. Since the P1 and P2 promoters are overlapping, all of the fusions retained part of the −10 sequence of P2; removing it would alter the GraL sequence. Therefore, as control, a similar transcriptional fusion to UP-p*lac*_UV5_-*greA*-*lacZ* but bearing only the first 10 nt of the *greA* leader was also constructed, to exclude the possibility that the P2 promoter still remained active and was prone to GreA regulation (UP-p*lac*_UV5_-*greA*+*10*-*lacZ)*. Importantly, all constructs lack the p*lac* operator region and thus are not under LacI control. The constructs were introduced in a single copy on the chromosome of a Δ*lacZ* strain (CF15617), and their activity was assessed by β-galactosidase activity assays in the presence or absence of GreA, overproduced from a low copy plasmid (a pGB2 derivative) under an inducible p*tac* promoter (pHM1873, called here pGreA; this plasmid does not encode GraL). pHM1883 served as the vector control (pV).

The results are presented in Figure 3 (middle and right panels). It is clear that GreA-mediated inhibition occurs in all three cases where GraL and its downstream region are present (Figure 3a–d), regardless of the promoter region present. The extent of inhibition depends on the presence of isopropyl-β-D-thio-galactopyranoside (IPTG)—only a minor effect is observed when compared to full downregulation that occurs upon 1 mM IPTG addition (at OD_600_ ~ 0.5-, 1.0- and 2.0, the fold repression by GreA was calculated to be - for p*greA*_all_-*lacZ*: 2.5-, 3.7-, and 4.9-fold when compared to the pV+IPTG strain (no IPTG: 1.2, 1.1, and 1.2, when compared to the pV strain); for p*lac*_UV5_-*greA*-*lacZ* fusion: 2.2-, 3.8- and 4.3-fold (no IPTG: 1.1, 1.2, and 1.6); and for UP-p*lac*_UV5_-*greA*-*lacZ*: 2.2-, 2.3-, and 2.8-fold (no IPTG: 1.5, 1.4, and 1.4)). This is probably due to the fact that native *greA* was also present on the chromosome and thus overproduction was needed to observe a substantial effect. It should be noted though that addition of IPTG also had a minor effect on the pV strains.

The only fusion that remained largely unaffected was the control bearing only the +10 *greA* leader region (similar β-galactosidase activities for strains bearing pGreA and pV in the presence of IPTG). Altogether, these data independently verify that GreA regulation of *greA* expression is independent of the promoter region but does depend on the *greA* leader.

### 2.3. The Amount of GraL Produced Depends on the GreA Level in the Cell

Next, we investigated whether the observed GreA-mediated transcriptional inhibition might be due to an increased production of GraL encoded in the *greA* leader region. As mentioned above, GraL terminates ~2/3 of transcripts that initiate in the p*greA* P1P2 promoter region, allowing only ~1/3 of transcripts to extend into full *greA* mRNA. We reasoned that increased transcriptional termination at the GraL terminator would thus yield less full-length transcripts and cause a decrease in the observed β-galactosidase activity of the employed fusions.

**Figure 4:**
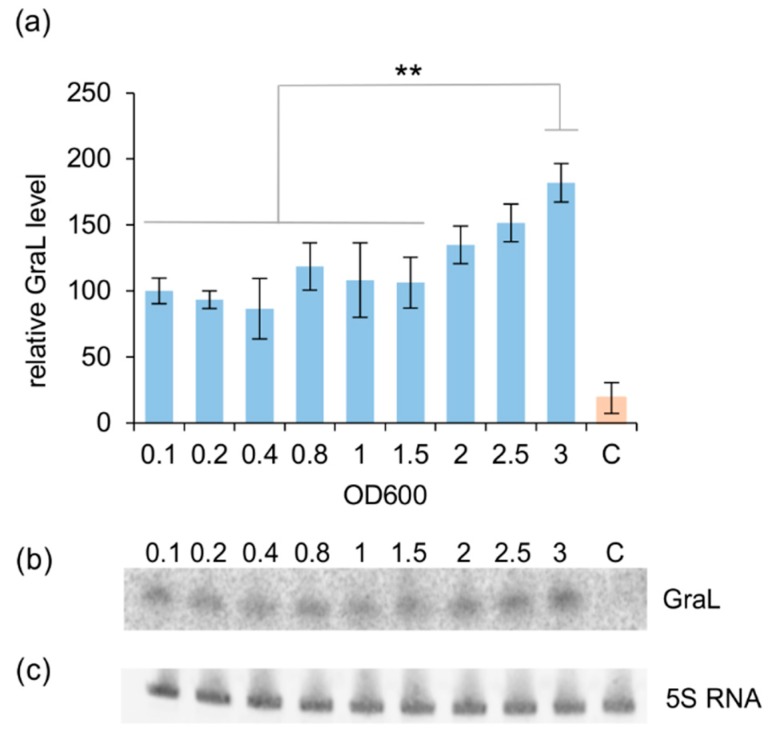
GraL levels monitored throughout bacterial growth. (**a**) GraL levels established by Northern blot analysis at OD_600_′s indicated; data were normalized to 5S RNA levels; the Δ*lacZ* Δ*greA* Δ*GraL* strain (ECMZ1604) served as control, the sample was taken at OD_600_ ~ 0.9 [C—in orange]. Growth of *E. coli* Δ*lacZ* (CF15617) was carried out in LB at 32 °C with aeration. Experiments were done in triplicate. Error bars represent S.D. Calculated *p*-values are indicated (** *p* < 0.005); (**b**) representative image of Northern blot with GraL visualized; (**c**) representative image of Northern blot with 5S RNA visualized.

First, we wanted to establish whether the GraL level is constant throughout the bacterial growth cycle or if it changes. Therefore, GraL levels were monitored by Norther blot analysis, employing the *E. coli* Δ*lacZ* strain (CF15617). As shown in Figure 4, the GraL level is rather constant in the log phase; however, it increases in the stationary phase by 1.8-fold at the highest OD_600_ tested, i.e., 3.0, when compared to the GraL level at OD_600_ = 0.1.

**Figure 5:**
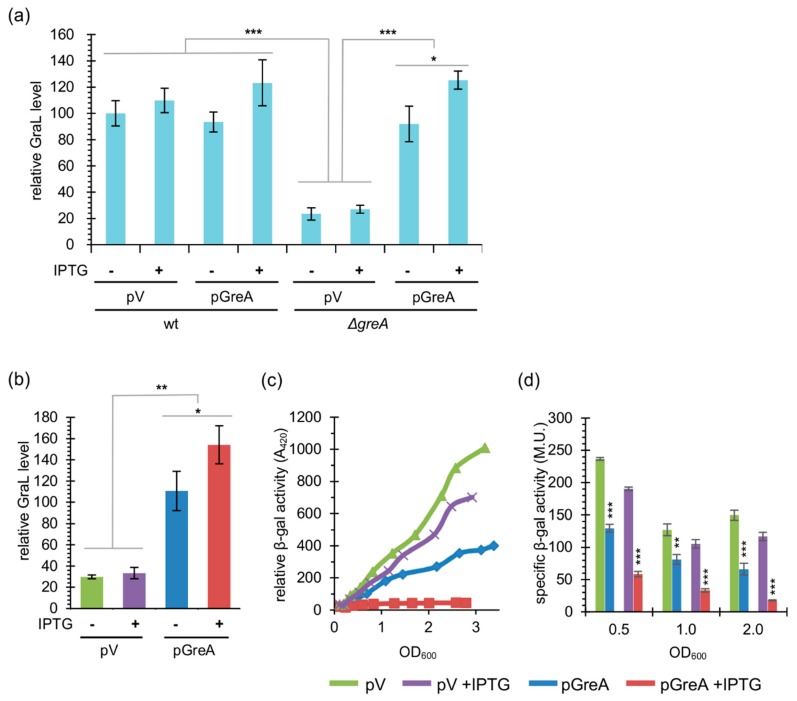
Increased GreA levels correlate with an increase in GraL abundance and a decrease in transcripts that are not terminated at the GraL terminator. (**a**) Relative GraL level was assessed by Northern blot analysis, as described in Figure 4. If present, IPTG was added to 1 mM at OD_600_ ~ 0.1. Samples were taken from wt or Δ*greA* strains at OD_600_ ~ 3.0. Data were normalized to wt/pV; this experiment was done in triplicate. (**b**) Same as in (**a**) but with the Δ*lacZ* Δ*greA* Δ*GraL* strain carrying the UP*lac*-*plac_UV5_*-*greA-lacZ* fusion; data were normalized to the pGreA sample; experiment was done in triplicate. (**c**) A representative differential plot of β-galactosidase activities of the strain used in (**b**). (**d**) Specific β-galactosidase activities at OD_600_ ~ 0.5, 1.0, and 2.0 of the strains presented in (**b**) and (**c**); this experiment was done in triplicate. Error bars represent SD. For clarity, *p*-values calculated only for pV *vs*. pGreA, and pV+IPTG *vs*. pGreA+IPTG are shown in (**d**). * *p* < 0.05, ** *p* < 0.005, *** *p* < 0.0005.

We next asked if the amount of GreA affects GraL abundance. Therefore, we measured the level of GraL produced in the wild type Δ*lacZ* and Δ*greA* Δ*lacZ* strains in the presence of pGreA or the control plasmid (pV). As demonstrated in Figure 5a, induction of GreA overexpression in wt/pGreA cells by adding 1 mM IPTG moderately increased the GraL level (a ~1.25-fold increase) when compared to IPTG untreated cells or cells carrying vector control, although this increase is not statistically significant. In contrast, in the Δ*greA* strains carrying pV, the GraL level decreased ~4-fold when compared to the wild type strain. When pGreA plasmid was now introduced, in the absence of IPTG, the GraL level has increased ~4-fold, almost reaching the level present in the wt/pV cells. When IPTG was added to 1 mM to further induce GreA overexpression, the GraL level in these cells reached the level present in the wt/pA + IPTG cells. These results strongly suggest that increasing the GreA level indeed has a positive effect on GraL production.

Next, we wanted to verify that the observed changes in GraL abundance correlate with our previous observations made with promoter region replacement fusions. The single copy UP*lac*-*plac_UV5_*-*greA*-*lacZ* fusion was chosen for these studies. However, this time, the strain was deleted for *greA* and GraL on the chromosome (ECMZ1604). In this setting, GraL originates only from the fusion construct under investigation. Figure 5b displays the GraL levels assessed by Northern blot analysis, whereas Figure 5c,d shows β-galactosidase reporter activity of the fusion corresponding to the full-length transcripts that were able to proceed through the GraL terminator and yield *lacZ* mRNA. It is evident that high GraL levels in the strain carrying pGreA plasmid correlate with decreased β-galactosidase activity of the tested fusion.

### 2.4. The Observed GreA Inhibition of greA Expression is not Due to GraL Acting in trans on its Own mRNA

Next, we wanted to investigate a possibility that GraL acting *in trans*, instead of GreA, enhances termination at the GraL terminator and thus increases GraL production. Therefore, similar experiments to those presented in Figure 3 were performed, where β-galactosidase activity of different *lacZ* fusions was monitored, but this time GraL (and not GreA) overproduction was induced from a low copy plasmid. No major differences in the activity of the fusions were noted, either in the wt or Δ*greA* Δ*GraL* background (Figure S1 and Table S1).

In addition, since sRNAs acting *in trans* often require Hfq protein for binding to their mRNA targets [5], β-galactosidase activity of the investigated fusions was also monitored in strains either overproducing Hfq or carrying an *hfq* deletion (again, GreA levels were not altered). The only substantial difference was observed for the Δ*hfq* strain carrying the *pgreAall–lacZ* fusion, whose activity decreased about 1.5-fold in comparison to the wt strain throughout growth (Figure S2 and Table S2). This could imply that another sRNA could perhaps regulate *greA* transcription at the promoter (either directly or indirectly through another target), but GreA regulation is not involved in this step. When GreA overproduction was induced from the pGreA plasmid in Δ*hfq* strains, similar behavior to that reported in Figure 3 was observed for all fusions (Figure S3 and Table S3). We take this to mean that GreA autoregulation does not require Hfq.

### 2.5. GreA Mediated Regulation Requires Cis-Acting Gral Sequences in Addition to the Terminator Structure

**Figure 6:**
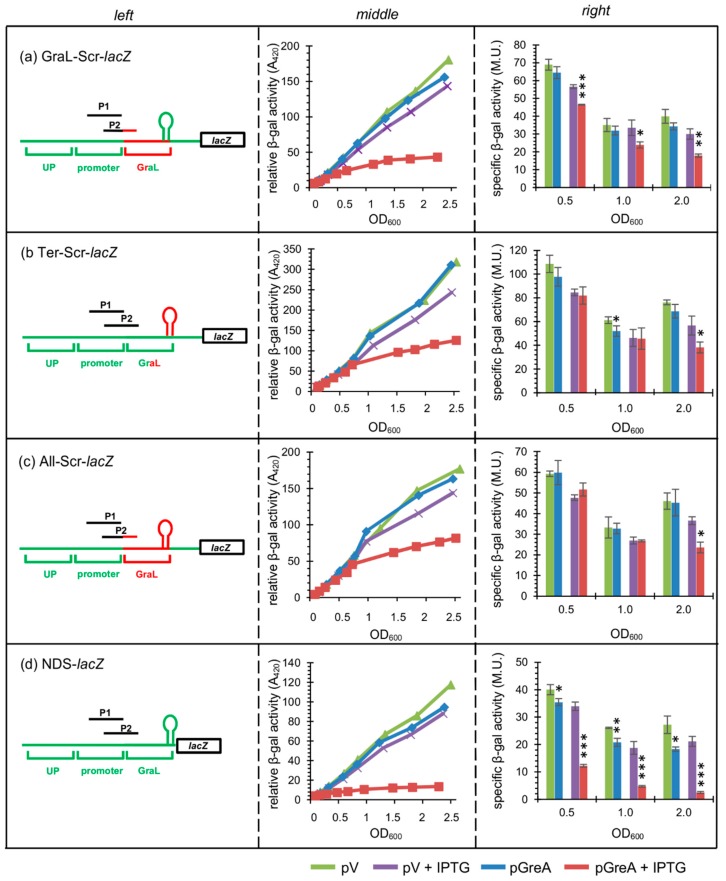
The GraL sequence (both upstream of and forming the terminator hairpin) determines autoregulation exerted by GreA. Assessment of β-galactosidase activity in strains overproducing GreA from a multicopy plasmid (pGreA) for: (**a**) GraL–Scr–*lacZ* (region from +1 to +18 scrambled); (**b**) Ter–Scr–*lacZ* (terminator hairpin arms switched with each other); (**c**) All–Scr–*lacZ* ((a) and (b) combined); (**d**) NDS–*lacZ* (“no downstream region”) fusions. Wt Δ*lacZ* (CF15617) strains carrying appropriate single copy fusions were grown in LB at 32 °C with aeration; if present, IPTG was added at OD_600_ ~ 0.1 to 1 mM; pV—vector control. Left panels—schematic representation of the *lacZ* fusions employed; hairpin symbolizes the GraL terminator; not drawn to scale. Middle panels—differential plots of β-galactosidase activities *vs.* OD_600_, following transcriptional activity throughout growth. The slope of each curve corresponds to the specific activity of β-galactosidase. Right panels—specific β-galactosidase activities assessed for each strain at OD_600_ ~ 0.5, 1.0, and 1.5 (experiment was done in triplicate). Error bars represent S.D. For clarity, *p*-values calculated for pV vs. pGreA, and pV+IPTG *vs*. pGreA+IPTG are only shown. * *p* < 0.05, ** *p* < 0.005, *** *p* < 0.0005.

The results obtained thus far seem to point to the *in cis* role of GraL. Alternatively, it could be that not the GraL sequence *per se*, but rather only the terminator structure participates in the GreA autoregulation, as our previous investigation had hinted that the terminator hairpin might be involved in this process [25]. To answer this question, another set of transcriptional fusions with the *lacZ* reporter gene were constructed (Figure 6, left panels).

First, the initial GraL sequence (+1 to +18) that does not include the terminator hairpin was randomly scrambled, preserving the GC (guanosine and cytosine) content (GraL–Scr*–lacZ* fusion). In this case, GreA overproduction from the pGreA plasmid still represses the full-length transcript formation as judged from β-galactosidase reporter activities, although to a much lesser degree (1.2-, 1.4-and 1.7- fold repression at OD_600_ = 0.5, 1.0, and 2.0, respectively, *vs*. 2.5, 3.7-, and 4.9-fold inhibition at the respective OD’s for the wt fusion, comparing Figure 6a with Figure 3a). Second, we switched the sequences of the terminator hairpin arms (region spanning +19 to +48) which retained base pairings to preserve the terminator’s secondary structure (Ter–Scr–*lacZ* fusion, Figure 6b). This resulted in similarly abolished GreA mediated repression in the logarithmic phase of growth, with only some inhibition in the late stationary phase (1.0-, 1.0-, and 1.5-fold inhibition at OD_600_ = 0.5, 1.0, and 2.0, respectively). Third, a construct combining both modifications was employed (+1 to +18 scrambled and +19 to +48 region switched; All–Scr–*lacZ* fusion, Figure 6c), and yielded similar results (0.9-, 1.0-, and 1.5-fold repression at OD_600_ = 0.5, 1.0, and 2.0, respectively). In order to make sure that the scrambled sequences were still functional in termination, *in vitro* transcription reactions were carried out with these templates and confirmed that indeed termination at the modified sequences still takes place (Figure S4).

Finally, to determine if the sequence downstream of the terminator hairpin might be important for GreA autoregulation, as it may possibly pair with GraL, a fusion devoid of the sequence downstream of the U-rich tail after the terminator was constructed (the region immediately downstream of +59 was deleted; “no downstream sequence”: NDS–*lacZ*). As evidenced in Figure 6d, the downstream sequence does not contribute to GreA autoregulation, since the β-galactosidase reporter activities were similar to those of the wt fusion (2.8-, 3.9-, and 8.4-fold inhibition at OD_600_ = 0.5, 1.0, and 2.0, respectively; compare Figure 6d with Figure 3a).

All of the above data indicated that it is not only the terminator structure of GraL, but also its sequence (both upstream of the terminator and participating in the terminator formation) that are important for GreA autoregulation. Thus, GraL acting *in cis* is necessary for GreA autoregulation to occur.

### 2.6. None of the Nus Factors nor σ^E^ Affect greA Regulation

Next, we wanted to assess whether Nus factors would have any effect on the GraL terminator read-through since they are known to affect transcriptional elongation, as well as termination and antitermination efficiency [1,28]. The four *lacZ* fusions with promoter region replacements shown in Figure 3a were tested in the absence or presence of plasmids carrying *nusA*, *nusB*, *nusE,* or *nusG* under an IPTG inducible p*tac* promoter. Wt Δ*lacZ* (CF15617) strains were used, expressing *greA* from the chromosome. No substantial effect was observed when any of the Nus factors were overproduced (Figure S5 and Table S4).

We also tested whether σ^E^ induction would have any effect on the four *lacZ* fusions tested, since the *greA* P2 promoter is σ^E^ dependent. We reasoned that GraL termination efficiency might also be under σ^E^ control, since it has been recently suggested that factors inducing a given sRNA transcription often also affect termination at their respective intrinsic terminators [29]. Here, in order to induce σ^E^, we used a pBAI66 plasmid overexpressing a misfolded peptide that accumulates in the periplasm [30]. Again, wt Δ*lacZ* (CF15617) strains were used as hosts. Our data indicated that the p*greA_all_-lacZ* fusion is only moderately responsive to σ^E^; however, we observed no differences in β-galactosidase activity for the other three constructs (Figure S6 and Table S5).

## 3. Discussion

In the study presented here, we turned to GreA, a global transcriptional regulator, shown to have many positive effects, such as rescuing stalled RNAP complexes and being responsible for proofreading and transcriptional fidelity [9,21,22]. GreA has been also shown to negatively influence the DNA break repair process [31]. It was also shown to influence gene expression of about 190 genes, when overproduced in *E. coli* (assuming significant changes in expression are at a log level of 0.5 [32]). On the other hand, competition between GreA and other factors that bind to the RNAP secondary channel is predicted to be perturbed when GreA levels change markedly [18]. One can thus imagine that there is a need for *greA* expression to be fine-tuned and tightly regulated.

In our earlier work, we showed that *greA* leader encodes a small RNA (GraL) and that GreA autoregulates its own gene expression [25]. Here, we demonstrate that this autoregulation depends on GraL acting *in cis*, i.e., the GraL sequence is necessary and sufficient for the full GreA-mediated repression to occur. Interestingly, the sequence upstream of the terminator is important, as well as the terminator hairpin sequence itself. The promoter region does not seem to play a major part here, indicating that GreA does not affect the transcription initiation step. In addition, catalytically active GreA is required for the autoregulation to take place. One prospect of future research may include investigation of GraL placement in the leader structure, to see if placing it further from the promoters would have any effect on autoregulation.

It has been suggested recently that the genes downstream of the sRNA’s terminators are expressed discordantly from the sRNAs encoded in their 5′UTR regions [29]. Given the results described above, this seems not to be the case for GraL and *greA*. In this instance, GreA levels tightly regulate GraL abundance, and GraL acts *in cis* to determine the resulting GreA levels. It is not surprising that such a tight regulatory circuit exists since, as mentioned above, GreA is an important transcription regulator and its levels remain fairly constant throughout growth, being slightly higher in the exponential phase of growth than in the stationary phase [33]. What is surprising in this context is that GraL is also acting *in trans*, with *nudE* (encoding a nudix-type hydrolase) being the most likely target [26]. This implies that the GreA regulatory web is even more widespread than previously appreciated. In addition, microarray experiments showed that GraL affects more than 100 genes when overproduced, and it was demonstrated that the presence of GraL enhances cellular fitness as well [25]. In addition, another screen revealed about 40 synthetic lethal genes with GraL, representing diverse functions [26].

GraL acting both *in cis* and *in trans* is a rare example of a dual action sRNA. To our knowledge, there is only one other well-documented example of this type—SreA riboswitch from *Listeria monocytogenes* [34], and one report implying such a possibility for Brt1 sRNA from *Bartonella hensenae* [35]. It is possible that such a dual mechanism of action might be more common than previously thought and more examples await detailed studies.

The simplest model of GreA autoregulation could be imagined to feature catalytically-active GreA as a factor inducing termination at the GraL terminator and thus regulating read-through of the full-length *greA* mRNA. However, it is intriguing that GreA autoregulation has been only observed so far *in vivo* and not *in vitro* ([25]; and Figure S4). This implies that GreA either requires an accessory factor to induce termination (direct mode of GreA autoregulation), or GreA is regulating expression of another factor that in turn regulates GraL termination (indirect mode of GreA autoregulation). Arguments can be made in favor of each model. The involvement of GreA antipause activity (Figure 2) can be seen as evidence of indirect regulation, where GreA could, for example, enhance gene expression of a *greA* repressor. At the same time, indirect regulation would require the existence of a binding site for such a repressor—in this case, perhaps the GraL sequence could serve as such a site, since it is essential for autoregulation to occur (Figure 3d). However, scrambling of the GraL sequence does not alleviate GreA repression (Figure 6c), and can be seen as evidence against the indirect model. On the other hand, the same arguments can be made in case of the direct model—requirement for GreA antipause activity and specific sequence binding cannot be excluded. Certainly, further studies are required to clarify the exact regulatory mechanism.

As already mentioned, it has been suggested that the efficiency of termination at the sRNA intrinsic terminators is regulated by the same signals (physiological or occurring at times of stress) that induce transcriptional initiation of a given sRNA [29]. This seems not to be true for GreA since here it does not affect the transcription initiation step. However, we wondered whether σ^E^ could be the auxiliary factor enhancing GreA’s activity, since σ^E^ controls transcription initiation from the p*greA* P2 promoter. Although we did observe a moderate increase in transcriptional activity of the p*greA_all_–lacZ* fusion *in vivo* when σ^E^ activity was induced by accumulation of misfolded proteins (GreA level was kept constant here), the activity of the other fusions did not increase when the promoter region was replaced with p*lac_UV5_*. Besides confirming that our basic fusion construct is responsive to σ^E^, this also implies that the auxiliary factor necessary for GreA autoregulation is not under σ^E^ control. We believe that Hfq and the Nus factors are also excluded as the possible accessory factors to GreA. Further studies, perhaps employing transposon random libraries or plasmid libraries of overexpressed genes, are needed to identify the additional factor necessary for GreA autoregulation to occur. Another approach could involve a random point mutant screen for *greA* leader isolates that block *in vivo* autoregulation, which could then be used in a pulldown search for a protein that binds to normal *greA* leader sequences but not to the autoregulation inefficient mutant. Additionally, another future approach could include investigating a possible role of Rho in this process. Although GraL is very short (only 19 nts before the terminator structure is formed) and terminates at a Rho-independent terminator, it cannot be excluded that the GraL sequence and structure may provide a conditional binding site for Rho, which together with GreA might enhance termination.

How has the GreA/GraL system developed? It seems more plausible that GreA autoregulation (GraL acting *in cis*) had evolved first, and GraL acting *in trans* arose as a secondary function. It should be noted that regulation by early transcriptional termination at the 5′UTR is a common mechanism among bacteria, where attenuators, riboswitches, or T-boxes are employed [36]. All of these mechanisms often rely on alternative RNA secondary structures that under certain conditions may form an intrinsic terminator.

Attempting to qualify GraL as an attenuator or a riboswitch is a complex issue. For one thing, our data show that some remnant GreA autoregulation still persists when either the sequence upstream of the terminator or the terminator hairpin sequence is scrambled, pointing to a slight degree of sequence identity independence. On the other hand, even though termination read-through was enhanced *in vivo*, termination was still observed when using these fusions as templates in *in vitro* transcription. This indicates that the β-galactosidase activities observed *in vivo* did reflect a change in response to the unknown accessory factor, or to GreA and the factor acting together, and did not merely result from abolished termination *per se*.

On the basis of classic attenuators, one would expect the effector to counteract or alleviate the “normal” (physiological) course of events, i.e., induce terminator formation if usually not formed, or prevent its formation if usually formed. In the case of GreA and GraL, this is not so—GreA (directly or indirectly) seems to amplify termination at the GraL terminator instead of abolishing it. Similar conundrums are encountered when thinking of GraL in terms of a riboswitch—the accessory molecule, acting jointly with GreA, should abolish termination, not enhance it. Of course, it cannot be excluded that under certain conditions GraL termination is overridden—such conditions remain yet to be found. It is also possible that GraL may act as an attenuator or riboswitch under a certain set of conditions, while GreA autoregulation forms a separate or possibly intertwining regulatory circuit.

In the simplest terms, the GreA/GraL system represents a feedback regulatory loop with many unique features. The *greA* expression is controlled by the GreA protein itself (through GraL acting *in cis*), but this system also widens GreA’s regulatory web (through GraL acting *in trans*). Definitely, many questions still remain and future studies are needed to dissect the precise mechanism governing *greA* expression. Our study is an example of how complex and intricate systems are in place to regulate and fine-tune the expression of a global regulator (in this case, a very important transcriptional factor). In addition, the results presented here may aid in the discovery of similar systems, which nevertheless may possess unique variations of their own.

## 4. Materials and Methods

### 4.1. Strains and Plasmids

All transcriptional fusions with *lacZ* were constructed by introducing an appropriate DNA fragment (obtained either by PCR or through a GeneArt (GeneStrings) service from ThermoFisher Scientific, Waltham, MA, USA) into a pRS415 plasmid (restriction sites: *Bam*HI/*Eco*RI) [37], except for the *greA* −1030 to +175 *lacZ* fusion, which was constructed by cloning the DNA fragment obtained by PCR (primers G1 and G7) into pRS515 plasmid [37]. Thus, the obtained multicopy fusions were introduced as a single copy on the *E. coli* chromosome with the use of phage λRS45 and verified to be monolysogens by PCR [38].

For GraL overproduction, pGraL plasmid was constructed by annealing MDGLUP and MDGLDWN primers to each other, followed by 1 cycle of extension by Taq polymerase (GoTaq, Promega, Madison, WI, USA) and subsequent dsDNA purification on Amicon Ultra 0.5 mL device (10 kD cut-off; Merck, Germany); this fragment was then digested with *Eco*RI/*Hind*III and cloned into a pGB2 vector derivative cut with the same enzymes. For GreA overproduction, pHM1873 plasmid (pGreA) was used, and pHM1883 was used as control (pV) [18]. For Nus factor overproduction, pNusA, pNusB, pNusE, and pNusG plasmids were used [39], with pHM1883 as control. For σ^E^ induction, pBAI66 was used, with pTrc99 as control [30,40]. For Hfq overproduction, F+hfq was used, with F+rpoB as control [41]. For the construction of pBR–GreA and pBR–GreA D41A, both alleles were amplified together with their native promoters (using G1 and G11 primers) from MG1655 [42] and TP1204 [43], respectively, and cloned into pBR322 [44] using *EcoR*I and *Pst*I restriction sites. Gene deletions/modifications were introduced by standard P1 transduction [45]. Strain JW4130-1 was used as a source of *Δhfq-722:kan* [46]. All strains and plasmids used are listed in Table S6 and Table S7, respectively. The oligonucleotides and synthetic DNA fragments used are listed in Table S8. All oligonucleotides were ordered from Sigma/Merck (Darmstadt, Germany), and all restriction enzymes were purchased from ThermoFisher Scientific (Waltham, MA, USA).

### 4.2. β-Galactosidase Assays

These assays were performed as described in [45].

### 4.3. Northern Blots

Northern blot analysis was carried out as described in [25] with modifications that allowed for the detection of chromosome encoded GraL. Briefly, RNA was isolated from cultures of growing cells (5 OD_600_ units were removed for sampling) with Trizol reagent (ThermoFisher Scientific, Waltham, MA, USA). Next, 20 µg of total RNA were loaded onto a 10% acrylamide TBE-Urea gels (ThermoFisher Scientific, Waltham, MA, USA), and run in 1× TBE buffer at 160 V for 58 min. Next, RNA was transferred onto a GeneScreen Plus membrane (Perkin Elmer, Waltham, MA, USA) with the use of Iblot2 Transfer Device (Life Technologies/ThermoFisher Scientific, Waltham, MA, USA) at 20 V for 3 min. The RNA was then crosslinked to the membrane by UV_254_ irradiation for 15 min, followed by baking at 80 °C for 1 h. The membrane was then blocked in UltraHyb-Oligo buffer (ThermoFisher Scientific, Waltham, MA, USA) for 1 h at 42 °C, followed by the addition of P^32^-labeled probe to GraL (5′end-labeled pAp2sRNA primer; 1.2 μCi/mL final concentration), and Cy3 labeled probe to 5S RNA (5′end-labeled 5SProbe primer [47]; 50 nM final concentration). The incubation was carried out at 42 °C for 16 h, and was followed by two 20 min washes with 2 × SSC, 0.5% SDS buffer at room temperature. The membrane was then directly scanned with the use of Typhoon scanner (GE Healthcare, Life Sciences, Pittsburgh, PA, USA) to visualize 5S RNA (excitation: 532 nm; emission: 580 nm), followed by exposition with appropriate Phosphoimager screen to visualize GraL and scanned with the same scanner in the Phosphoimager mode.

### 4.4. Single Round in vitro Transcription

This assay was basically performed as described in [25]. *E. coli* RNA polymerase was purified as described in [48]. Linear templates were obtained by PCR amplification of the appropriate DNA fragments based on the corresponding fusion constructs with the following primer pair: HMPr368 and pBplasdw (Table S8). GreA was purified as described in [16].

### 4.5. Statistical Analysis

Statistical analysis was carried out either with the use of Microsoft Excel 2013 (Microsoft Corporation, Redmond, WA, USA) or Statistica 13.1 (StatSoft Polska, Cracow, Poland) software. Student’s two-tailed t-test was employed for Figure 3, Figure 5d and Figure 6, while one-way Anova with post-hoc Tukey test was used to evaluate data presented in Figure 2, Figure 4 and Figure 5a,b. Differences were deemed as statistically significant for *p* < 0.05.

## Supplementary Materials

Supplementary materials can be found at https://www.mdpi.com/1422-0067/20/20/5224/s1.

## Abbreviations

IPTG	Isopropyl-β-D-thio-galactopyranoside
RNAP	RNA polymerase
sRNA	small RNA
UTR	untranslated region
nt	nucleotides
wt	wild type
