# Supplementary material for: Autoregulation of greA Expression Relies on GraL Rather than on greA Promoter Region
Source: Int J Mol Sci. 2019 Oct 22;20(20):5224. doi: 10.3390/ijms20205224 (PMC6829880; doi:10.3390/ijms20205224)
Supplement: Supplementary file 1 [file ijms-20-05224-s001.zip › ijms-616649-supplementary.pdf]

Autoregulation of *greA* Expression Relies on GraL Rather than on *greA* Promoter Region

Maciej Dylewski, Llorenç Fernández-Coll, Bożena Bruhn-Olszewska, Carlos Balsalobre and Katarzyna Potrykus

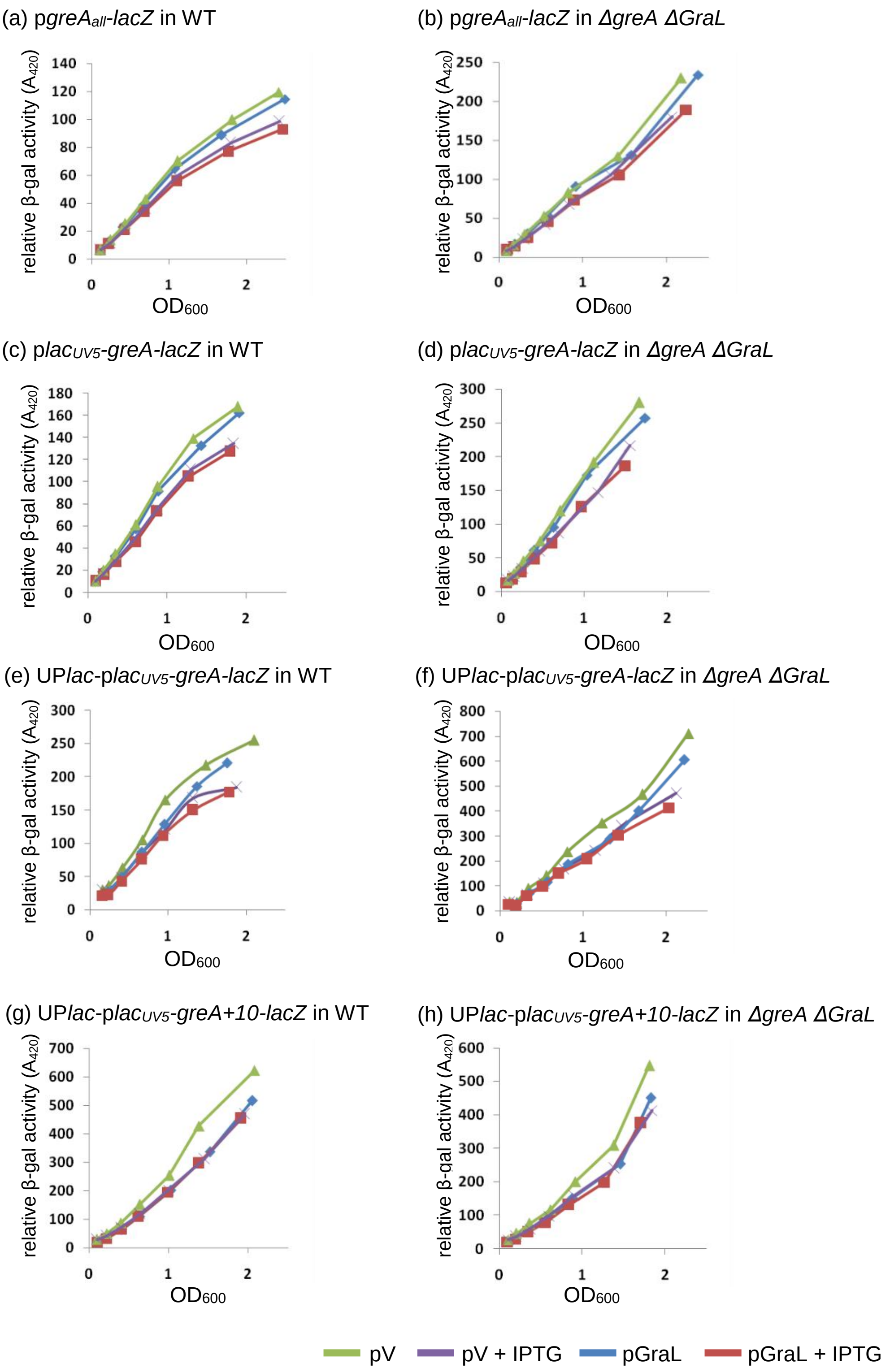

Figure S1. GraL does not affect *greA* expression when supplied *in trans*. Differential plots of beta-galactosidase activity of four *greA*-promoter/leader region fusions with *lacZ* vs OD_{600}, in the presence

or absence of GraL overproduced from a multicopy plasmid (pGraL; a pGB2 derivative). When present, IPTG was added to 1 mM at OD_{600}~0.1. (a), (c), (e) and (g) – wt (*ΔlacZ*) (CF15617) strains were used; (b), (d), (f) and (h) - *ΔlacZ ΔgreA ΔGraL* (ECMZ1604) strains were used. pV- vector control (pHM1883). Experiments were done with one biological replicate and 6-10 technical replicates; calculated specific activities are presented in Table S1.

Table S1. Calculated β -galactosidase specific activity for the four *greA-lacZ* fusions in strains overproducing GraL from a multicopy plasmid (pGraL). pV – vector control. Average values were calculated from plots presented in Figure S1, based on at least 3 points where the β -galactosidase activity values were roughly linear. S.D. is given in parenthesis.

	pgreA$_{\text{all}}$-l*acZ*	p*lac*$_{\text{UV5}}$-*greA-lacZ*	UP$_{lac}$-*plac*$_{\text{UV5}}$-*greA-lacZ*	UP$_{lac}$-*Plac*$_{\text{UV5}}$-*greA+10- lacZ*
WT strains				
pV	60.25 (+/- 3.33)	103.74 (+/- 3.52)	155.65 (+/- 9.39)	226.39 (+/- 9.31)
pV +IPTG	50.74 (+/- 2.89)	84.38 (+/- 2.38)	125.09 (+/- 3.01)	183.29 (+/- 4.69)
pGraL	57.37 (+/- 2.75)	97.07 (+/- 4.55)	128.89 (+/- 6.75)	164.31 (+/- 8.10)
pGraL+IPTG	49.08 (+/- 3.00)	80.12 (+/- 3.14)	110.20 (+/- 8.80)	160.85 (+/- 14.27)
Δ*graL*Δ*greA* strains				
pV	94.60 (+/- 3.51)	169.54 (+/- 5.16)	275.89 (+/- 17.53)	210.95 (+/- 14.13)
pV +IPTG	77.33 (+/- 3.20)	132.62 (+/- 8.63)	210.64 (+/- 7.65)	170.21 (+/- 6.85)
pGraL	89.49 (+/- 6.33)	158.76 (+/- 8.46)	213.80 (+/- 13.42)	166.75 (+/- 8.16)
pGraL+IPTG	75.82 (+/- 3.19)	126.19 (+/- 6.60)	199.76 (+/- 9.46)	150.89 (+/- 6.93)

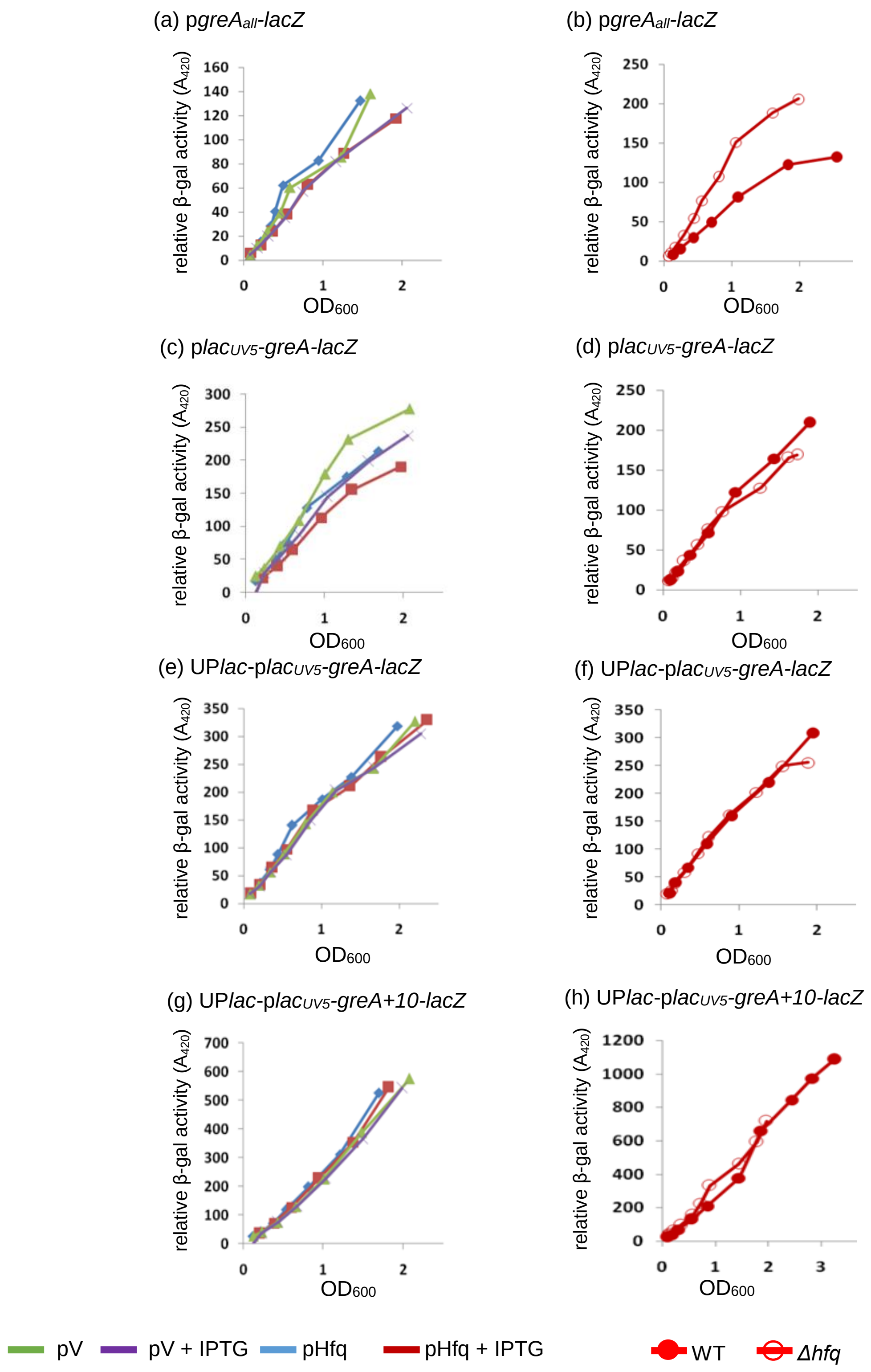

Figure S2. An *hfq* deletion de-represses the p*greA*$_{all}$-*lacZ* fusion's activity, but Hfq has no effect on other fusions where the promoter region has been replaced with p*lac*$_{UV5}$. Differential plots of β-galactosidase activity of four *greA*-promoter/leader region fusions with *lacZ* vs OD_{600}. Fusions used are indicated at the top of each panel. (a), (c), (e) and (g) in the presence or absence of Hfq overproduced from a multicopy plasmid (pHfq; an F+ derivative with *hfq* under a p*tac* promoter).

When present, IPTG was added to 1 mM at OD$_{600}$~0.1; wt *ΔlacZ* (CF15617) strains were used; pV- vector control (F+ *rpoB*). (b), (d), (f) and (h) – either wt *ΔlacZ* or *ΔlacZ Δhfq* strains were used. Experiments were done with one biological replicate and 6-10 technical replicates; calculated specific activities are presented in Table S2.

Table S2. Calculated β -galactosidase specific activity for the four *greA-lacZ* fusions in strains overproducing Hfq from a multicopy plasmid (pHfq) or in a *Δhfq* strain. pV – vector control. Average values were calculated from plots presented in Figure S2, based on at least 3 points where the β - galactosidase activity values were roughly linear. S.D. is given in parenthesis.

	p*greA*$_{all}$-*lacZ*	p*lac*$_{UV5}$-*greA-lacZ*	UP*lac*-*Plac*$_{UV5}$-*greA-lacZ*	UP$_{lac}$-*Plac*$_{UV5}$-*greA+10- lacZ*
pV	78.29 (+/- 7.49)	166.68 (+/- 10.47)	143.83 (+/- 5.69)	271.20 (+/- 8.58)
pV +IPTG	69.59 (+/- 5.65)	128.88 (+/- 6.01)	129.71 (+/- 3.91)	259.83 (+/- 10.99)
pHfq	83.89 (+/- 9.02)	135.40 (+/- 9.62)	161.21 (+/- 2.65)	240.27 (+/- 18.04)
pHfq+IPTG	70.56 (+/- 5.24)	108.98 (+/- 7.51)	147.43 (+/- 7.17)	257.55 (+/- 5.55)
WT	68.44 (+/- 2.18)	120.93 (+/- 6.92)	186.16 (+/- 9.03)	234.60 (+/- 9.53)
Δhfq	106.69 (+/-5.02)	133.30 (+/- 5.12)	196.73 (+/- 3.44)	283.66 (+/- 14.11)

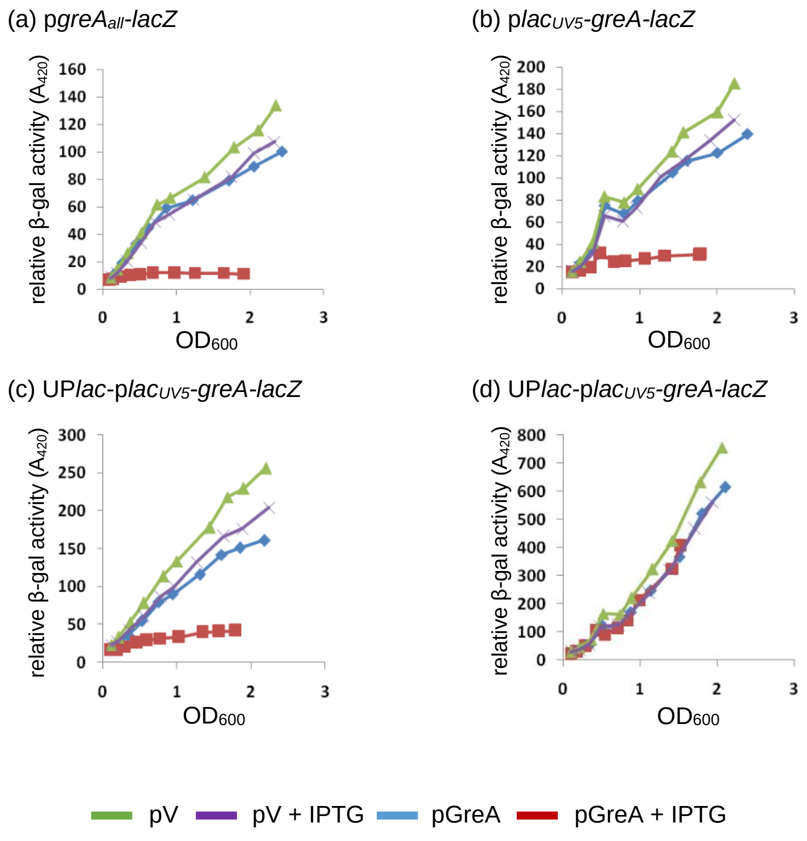

Figure S3. An *hfq* deletion does not alter GreA mediated regulation of *greA* expression. Differential plots of β-galactosidase activity of four *greA*-promoter/leader region fusions with *lacZ* vs OD$_{600}$, in the presence or absence of GreA overproduced from a multicopy plasmid (pGreA). (a) p*greA*$_{all}$-*lacZ; (b)* p*lac*$_{UV5}$-*greA-lacZ; (c)* UP-p*lac*$_{UV5}$-*greA-lacZ*; (d) UP-p*lac*$_{UV5}$-*greA+10-lacZ* fusions. When present, IPTG was added to 1 mM at OD$_{600}$~0.1; *ΔlacZ Δhfq* strains were used; pV- vector control. Experiments were done with one biological replicate and 6-10 technical replicates; calculated specific activities are presented in Table S3.

Table S3. Calculated β-galactosidase specific activity for the four *greA-lacZ* fusions in *Δhfq* strains overproducing GreA from a multicopy plasmid (pGreA). pV – vector control. Average values were

calculated from plots presented in Figure S3, based on at least 3 points where the β -galactosidase activity values were roughly linear. S.D. is given in parenthesis.

	p*greA*$_{all}$-l*acZ*	p*lac*$_{UV5}$-*greA*-*lacZ*	UP*lac*-*Plac*$_{UV5}$-*greA*-*lacZ*	UP$_{lac}$-*Plac*$_{UV5}$-*greA*+*10*- *lacZ*
pV	68.41 (+/- 12.24)	92.57 (+/- 4.44)	131.92 (+/- 6.86)	248.16 (+/- 30.41)
pV +IPTG	57.25 (+/- 9.40)	77.15 (+/- 2.52)	105.28 (+/- 4.20)	191.36 (+/- 20.02)
pGreA	59.99 (+/- 12.30)	78.12 (+/- 6.54)	94.43 (+/- 7.62)	190.56 (+/- 25.44)
pGreA+IPTG	12.36*	22.49*	32.38*	164.21 (+/- 5.04)

* β -galactosidase specific activity was calculated at OD$_{600}$~1.0.

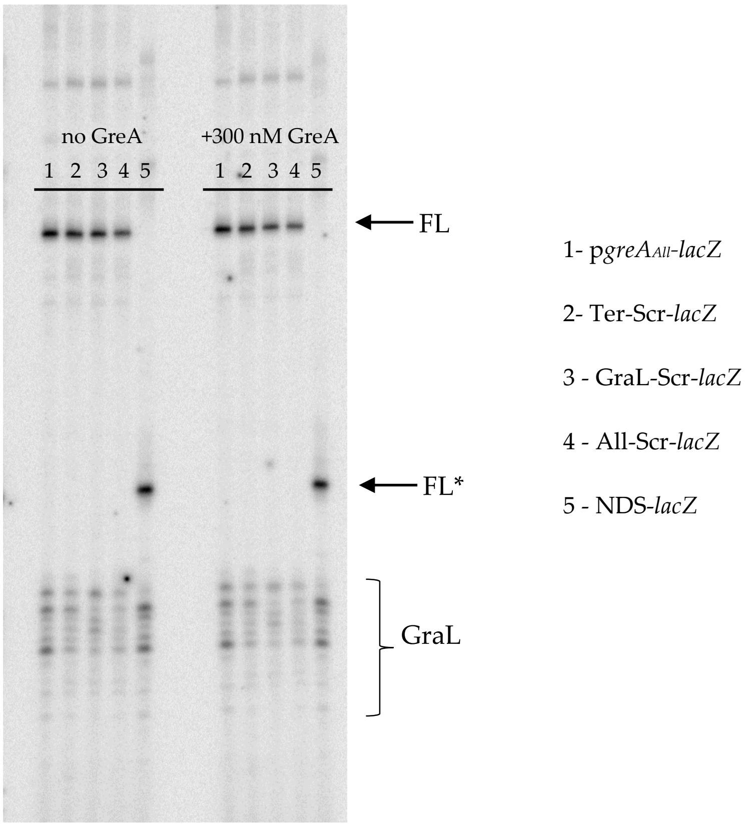

Figure S4. Constructs used for testing GraL leader/terminator region *in vivo* still undergo transcription termination *in vitro* regardless of absence or presence of GreA. Single round *in vitro* transcription was carried out on linear templates as described in the Materials and Methods section, in presence or absence of 300 nM GreA. FL indicates full-length runoff product formed; FL* - full-length runoff product on the shortened (NDS-*lacZ*) template; GraL – short, prematurely terminated transcripts. Templates used are indicated (diagrams of the constructs used are presented in the left panels in Figure 3a and Figure 6a-d). Experiments were done thrice.

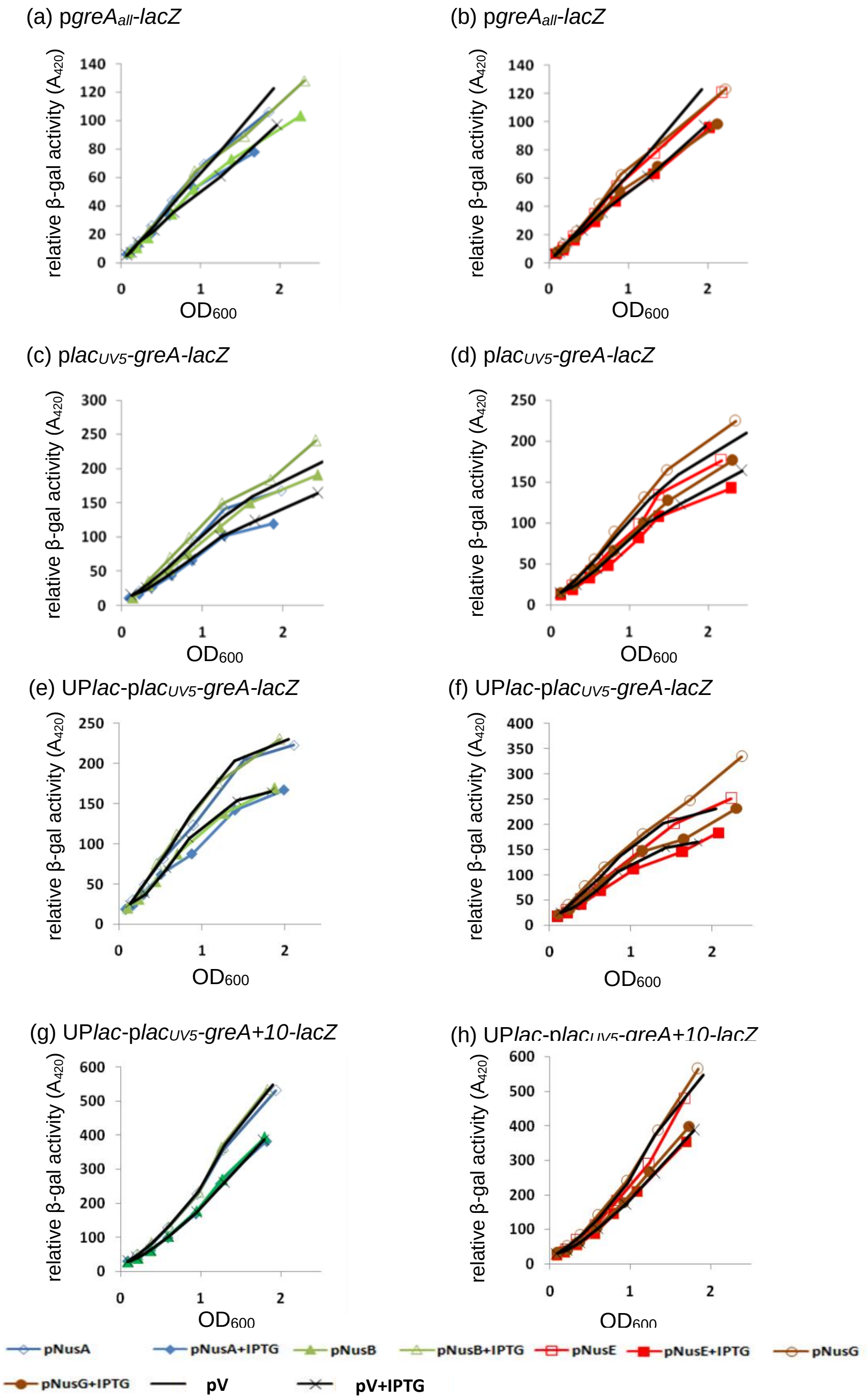

Figure S5. Nus factors do not alter *greA* expression. Differential plots of β-galactosidase activity of four *greA*-promoter/leader region fusions with *lacZ* vs OD_{600}, in the presence or absence of plasmids overproducing Nus factors. (a), (c), (e) and (g) – the effect of NusA or NusB was assessed; (b), (d), (f) and (h) – the effect of NusE or NusF was assessed. When present, IPTG was added to 1 mM at OD_{600}~0.1; wt *ΔlacZ* (CF15617) strains were used; pV- vector control. Fusions used are indicated at the top of each panel. Experiments were done with one biological replicate and 6-10 technical replicates; calculated specific activities are presented in Table S4.

Table S4. Calculated β -galactosidase specific activity for the four *greA-lacZ* fusions in strains overproducing Nus factors (NusA, NusB, NusG or NusE) from a multicopy plasmid (pNusA, pNusB, pNusG or pNusE; pGB2 derivatives). pV – vector control (pHM1883). Average values were calculated from plots presented in Figure S5, based on at least 3 points where the β -galactosidase activity values were roughly linear. S.D. is given in parenthesis.

	greA$_{all}$-*lacZ*	P*lac*$_{UV5}$-*greA-lacZ*	UP$_{lac}$-P*lac*$_{UV5}$-*greA-lacZ*	UP$_{lac}$-P*lac*$_{UV5}$-*greA+10- lacZ*
pV	65.70 (+/- 4.54)	99.47 (+/- 2.71)	146.45 (+/- 20.09)	238.53 (+/- 28.90)
pV+IPTG	57.71 (+/- 7.30)	77.78 (+/- 2.71)	111.80 (+/- 13.58)	183.73 (+/- 14.04)
pNusA	69.23 (+/- 3.41)	98.08 (+/- 8.20)	153.29 (+/- 18.14)	234.34 (+/- 27.57)
pNusA+IPTG	57.93 (+/- 3.26)	74.91 (+/- 4.10)	118.05 (+/- 17.40)	182.31 (+/- 17.11)
pNusB	65.34 (+/- 4.90)	112.09 (+/- 8.33)	150.08 (+/- 18.51)	239.36 (+/- 29.81)
pNusB+IPTG	54.59 (+/- 1.89)	91.34 (+/- 2.90)	113.81 (+/- 15.03)	184.55 (+/- 17.96)
pNusE	60.78 (+/- 1.92)	87.41 (+/- 6.96)	136.56 (+/- 5.68)	219.10 (+/- 14.86)
pNusE+IPTG	51.30 (+/- 1.97)	70.76 (+/- 5.26)	104.88 (+/- 8.98)	176.34 (+/- 14.02)
pNusG	66.18 (+/- 3.54)	106.43 (+/- 5.62)	164.05 (+/- 13.54)	247.76 (+/- 25.71)
pNusG+IPTG	54.91 (+/- 3.16)	83.01 (+/- 2.51)	124.96 (+/- 12.55)	190.84 (+/- 16.21)

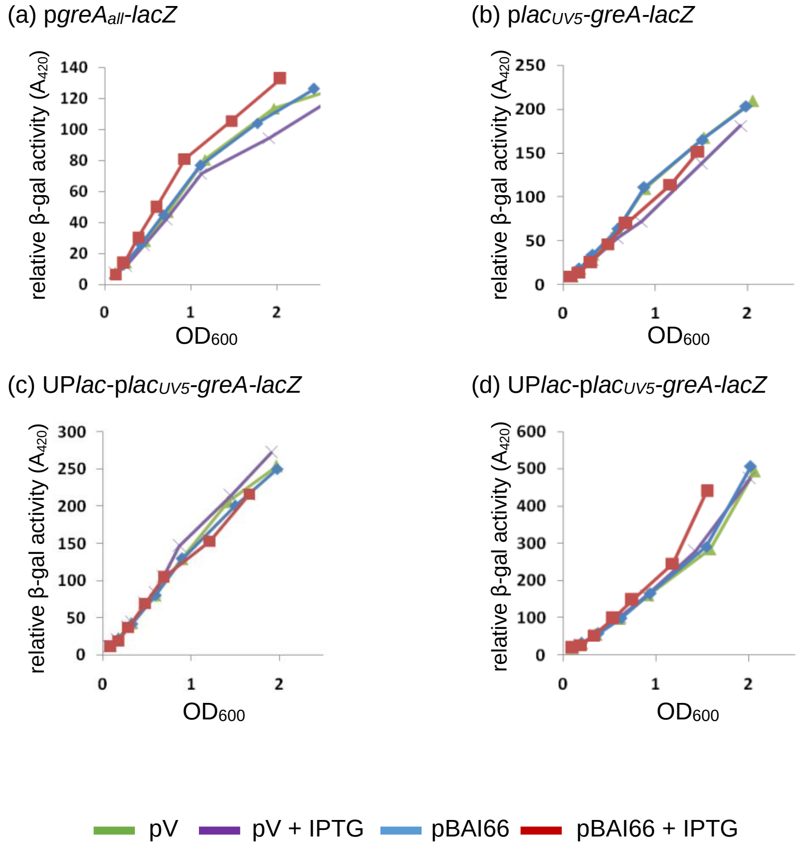

Figure S6. σ^E induction has a moderate effect on the *greA* P2 promoter but has no effect on transcription proceeding through the *greA* leader region. Differential plots of β-galactosidase activity of four *greA*-promoter/leader region fusions with *lacZ* vs OD$_{600}$, in the presence or absence of a misfolded peptide accumulation; the YYF peptide was overproduced from a multicopy plasmid (pBAI66). (a) p*greA*$_{all}$-*lacZ; (b)* p*lac*$_{UV5}$-*greA-lacZ; (c)* UP-p*lac*$_{UV5}$-*greA-lacZ*; (d) UP-p*lac*$_{UV5}$-*greA+10-lacZ* fusions. When present, IPTG was added to 1 mM at OD$_{600}$~0.1; wt *ΔlacZ* (CF15617) strains were used; pV- vector control (pTrc99). Experiments were done with one biological replicate and 6-10 technical replicates; calculated specific activities are presented in Table S5.

Table S5. Calculated β -galactosidase specific activity for the four *greA-lacZ* fusions in strains overproducing a misfolded YYF peptide, whose accumulation induces σ^E activity (pBAI66 plasmid, a pTrc99 derivative). pV – vector control (pTrc99). Average values were calculated from plots presented in Figure S6, based on at least 3 points where the β -galactosidase activity values were roughly linear. S.D. is given in parenthesis.

	greA_{all}-lacZ	P*lac*UV5-*greA-lacZ*	UP*lac*-*Plac*UV5-*greA-lacZ*	UP*lac*-*Plac*UV5-*greA+10- lacZ*
pV	60.51 (+/- 2.85)	106.51 (+/- 3.96)	131.69 (+/- 2.55)	162.29 (+/- 0.87)
pV+IPTG	54.01 (+/- 5.36)	89.80 (+/- 2.11)	131.53 (+/- 11.07)	158.19 (+/- 6.89)
pBAI66	61.46 (+/- 4.51)	108.03 (+/- 1.05)	128.56 (+/- 5.90)	161.60 (+/- 6.25)
pBAI66+IPTG	74.36 (+/- 11.14)	89.04 (+/- 6.89)	128.11 (+/- 18.15)	166.08 (+/- 18.54)

Table S6. Strains used in this work.

**Strain**	**Genotype**	**Source**
MG1655	F-, *ilvG*, *rph1*	Jin and Gross, 1988
AAG1	MG1655 Δ*lacZ*	Aberg et al. 2008
CF15617	MG1655 Δ*lacZ*	Vinella et al., 2012
ECMZ1601	CF15617 Δ*greA::cat*	Dylewski et al., 2018
ECMZ1604	CF15617 Δ*GraL* Δ*greA::cat*	Dylewski et al., 2018
JW4130-1	F-, *Δ(araD-araB)567*, *ΔlacZ4787*(::rrnB-3), *λ*$^-$, *rph-1*, *Δ(rhaD-rhaB)568*, *Δhfq-722::kan*, *hsdR514*	Baba et al., 2006
KP517	CF15617 (λ *greA*all-*lacZ*)	This work
KP623	CF15617(λ p*lac*UV5-*greA-lacZ*)	This work
KP625	CF15617 (λ UP*lac*-p*lac*UV5-*greA-lacZ*)	This work
LFC3	AAG1 (λ greA -1030 to +175-lacZ)	This study
LFC4	AAG1 greA::Cm (λ greA -1030 to +175-lacZ)	This study
MD324	CF15617 (λ UP*lac*-*Plac*UV5-*greA+10- lacZ*)	This work
MD404	KP517 *Δhfq-722::kan*	This work
MD405	KP625 *Δhfq-722::kan*	This work
MD406	KP623*Δhfq-722::kan*	This work
MD407	MD324 *Δhfq-722::kan*	This work
MD441	CF15617 (λ NDS-*lacZ*)	This work
MD442	CF15617 (λ GraL-Scr-*lacZ*)	This work
MD443	CF15617 (λ Ter-Scr-*lacZ*)	This work
MD444	CF15617 (λ All-Scr-*lacZ*)	This work
MD474	KP517 Δ*graL*Δ*greA::cat*	This work
MD475	KP623 Δ*graL*Δ*greA::cat*	This work
MD476	MD324 Δ*graL*Δ*greA::cat*	This work
TP1204	MG1655 *greA* D41A	Poteete, 2011

Table S7. Plasmids used in this work.

**Plasmid**	**Description**	**Source**
pBR322	*ori* pMB1, Ap^R, Tc^R	Bolivar et al., 1977
pBR-greA	*greA* with native promoter cloned into pBR322	This work
pBR-greA D41A	*greA* D41A with native promoter cloned into pBR322	This Work
pHM1786	pGB2 derivative, *ori* pSC101, *lacI*^{q}, Spc^R	[Vinella et al., 2012]
pGraL	GraL under p*tac* promoter cloned into pHM1786	This work
pGreA (pHM1873)	*greA* under p*tac* promoter cloned into pHM1786	[Vinella et al., 2012]
pV (pHM1883)	Vector control for pGreA and pGraL	[Vinella et al., 2012]

pRS415	For construction of transcriptional fusions with *lacZ* and subsequent transfer to λ and recombination on the chromosome; ApR	[Simons et al., 1987]
pRS551	pRS415 derivative, Ap^R Kan^R	[Simons *et al.*, 1987]
pKP-pAall	Region spanning -100 to +136 (relative to +1 of p*greA* P1 promoter) fused to *lacZ* in pRS415 at the *BamH*I/*Hind*III sites	[Potrykus et al., 2010]
pKP- $plac_{UV5}$-greA-lacZ	The same as pKP-pAall but promoter region replaced by $plac_{UV5}$	This work
pKP- UP_{lac}-$plac_{UV5}$-greA-lacZ	The same as pKP- $plac_{UV5}$-greA-lacZ but UP region replaced by region upstream of the native plac promoter; CRP binding site mutated (G->A at position -66, and C->T at position -55)	This work
pMD- UP_{lac}-*Plac*$_{UV5}$-*greA+10-lacZ*	The same as pKP- UP_{lac}-$plac_{UV5}$-greA-lacZ, but the region from +10 to +136 deleted	This work
pMD-GraL_Scr-*lacZ*	The same as pKP-pAall but the sequence from +1 to +18 randomly scrambled	This work
pMD- Ter_Scr-*lacZ*	The same as pKP-pAall but the sequence from +19 to +48 scrambled so as to switch the terminator hairpin arms with each other	This work
pMD- pAll_Scr-*lacZ*	The same as pMD- Ter_Scr but also with the sequence from +1 to +18 scrambled	This work
pMD-NDS-*lacZ*	The same as pKP-pAall but the region from +60 to +134 deleted	This work
pTrc99A	Vector, pBR322 ori, Ap^R	[Amann et al., 1988]
pBAI66	Plasmid for overproduction of a misfolded peptide (YYF) to induce σ^E; pTrc99A derivative	[Walsh et al., 2003]
pNusA	*nusA* cloned into pGB2	[Friedman et al., 1990]
pNusB	*nusB* cloned into pGB2	[Friedman et al., 1990]
pNusE	*nusE* cloned into pGB2	[Friedman et al., 1990]
pNusG	*nusG* cloned into pGB2	[Friedman et al., 1990]
F+rpoB	*rpoB* under p*tac* promoter in an F+ plasmid	[Saka et al., 2005]
F+hfq	*hfq* under p*tac* promoter in an F+ plasmid	[Saka et al., 2005]

Table S8. Oligonucleotides and synthetic DNA fragments used in this work.

**Name**	**Sequence (5'-3')**	**Description**
Oligonucleotides		
**pBplasdw**	CTTTCATCGGTTGTCGGATCC	For *in vitro* transcription template preparation, based on [Potrykus et al., 2010]
**G1**	GAGAATTCGCGATCATGTTGTCCGAC	For construction of pBR-GreA and pBR-GreA D41A plasmids, and construction of λ *greA -1030 to +175-lacZ* fusion

**G7**	CAGGATCCCGTAAGGTCATCGGAATAGC	For construction of λ *greA -1030 to +175-lacZ* fusion
**G11**	CACTGCAGCAACATCTTGAGTATTGGG	For construction of pBR-GreA and pBR-GreA D41A plasmids
**HMPr368**	CAGGAATTGGGGATCGGAATTC	For *in vitro* transcription template preparation, based on [Potrykus et al., 2010]
**KPr77**	TATGAATTCGGAAAGCGGGCAGTGAGCGCAACGCAATTAATGTAAGTTAGCTCATTCATTAGGCACCCCAGGCTTTACACTTTATGCTT	Forward primer to make the UP$_{lac}$-*Plac*$_{UV5}$-*greA+10- lacZ* fusion (with pKP- UP$_{lac}$-plac$_{UV5}$-greA-lacZ as template in the PCR reaction)
**KPr78**	TCTGGATCCACATTTTGATCCACACATTATACGAGCCGGAAGCATAAAGTGTAAAGCCTGGGGTGCCTAATGAATGAGCTAACTTACAT	Reverse primer to make the UP$_{lac}$-*Plac*$_{UV5}$-*greA+10- lacZ* fusion (with pKP- UP$_{lac}$-plac$_{UV5}$-greA-lacZ as template in the PCR reaction)
**MDGLDWN**	ATCAAGCTTAAGCAAAAAAATACCGACCCGGGTACAAGTCCCAGGTCAG	Reversed primer for GraL cloning (anneals to MDGLUP)
**MDGLUP**	ATGAATTCATCAAAATGTGAATTGTAGCTGACCTGGGACTTGTACCCG	Forward primer for GraL cloning (anneals to MDGLDWN)
**pAp2sRNA**	AAAATACCGACCGGGTACAAGTCCCAGGTCAGCTACAAT	Probe for P^{32} labeling; complementary to GraL (based on [Potrykus et al., 2010])
**5SProbe**	Cy3-CATGGGGTCAGGTGGGACCACCGCGCTACGGCCGCCAGGC	Cy3-labeled probe to 5S RNA (based on [Moll et al., 2003])
Synthetic DNA fragments [obtained through GeneStrings (GeneArt) service (Thermo)] *Bam*HI/ *Eco*RI sites are indicated		
**plac$_{UV5}$-greA**	TATGAATTCATAATCTCGCGCTAACAACCTGGAATCGAGCCGTCATACTACGGCGCAACGCCCTATAAAGTAAACGTTTACACTTTATGCTTCCGGCTCGTATAATGGTGGATCAAAATGTGAATTGTAGCTGACCTGGGACTTGTACCCGGGTCGGTATTTTTTTGCTTCTGGTCCCGGTAAGGAGTTATGCCGGGCAGGCCGAACAGCCGGGGTGGGTGAAGACTTGCCCTATCAGGAATATTCAGGATCC AGA	
**UP$_{lac}$-plac$_{UV5}$-greA**	TATGAATTCGGAAAGCGGGCAGTGAGCGCAACGCAATTAATGTAAGTTAGCTCATTCATTAGGCACCCCAGGCTTTACACTTTATGCTTCCGGCTCGTATAATGTGTGGATCAAAATGTGAATTGTAGCTGACCTGGGACTTGTACCCGGGTCGGTATTTTTTTGCTTCTGGTCCCGGTAAGGAGTTATGCCGGGCAGGCCGAACAGCCGGGGGGGTGAAGACTTGCCCTATCAGGAATATTCAGGATCCAGA	
**NDS_**	TATGAATTCCATAATCTCGCGCTAACAACCTGGAATCGAGCCGTCATACTACGGCGCAACGCCCTATAAAGTAAACGATGACCCTTCGGGAACTTCAGGGTAAAATGACTATCAAAATGTGAATTGTAG	

	CTGACCTGGGACTTGTACCCGGGTCGGTATTTTTTTGCTTGGATC CAGA
**GraL_Scr**	TATGAATTCCATAATCTCGCGCTAACAACCTGGAATCGAGCCG TCATACTACGGCGCAACGCCCTATAAAGTAAACGATGACCCTT CGGGAACTTCAGGGTAAAATGACTATAAGATATATGTACTAGG CTGACCTGGGACTTGTACCCGGGTCGGTATTTTTTTGCTTCTGGT CCCGGTAAGGAGTTATGCCGGGCAGGCCGAACAGCCGGGGTG GGTGAAGACTTGCCCTATCAGGAATATTCAGGATCCAGA
**Ter_Scr**	TATGAATTCCATAATCTCGCGCTAACAACCTGGAATCGAGCCG TCATACTACGGCGCAACGCCCTATAAAGTAAACGATGACCCTT CGGGAACTTCAGGGTAAAATGACTATCAAAATGTGAATTGTAT GGCTGGGCCCATGTTCAGGGTCCAGTCGATTTTTTTGCTTCTGGT CCCGGTAAGGAGTTATGCCGGGCAGGCCGAACAGCCGGGGTG GGTGAAGACTTGCCCTATCAGGAATATTCAGGATCCAGA
**All_Scr**	TATGAATTCCATAATCTCGCGCTAACAACCTGGAATCGAGCCG TCATACTACGGCGCAACGCCCTATAAAGTAAACGATGACCCTT CGGGAACTTCAGGGTAAAATGACTATAAGATATATGTACTAGT GGCTGGGCCCATGTTCAGGGTCCAGTCGATTTTTTTGCTTCTGGT CCCGGTAAGGAGTTATGCCGGGCAGGCCGAACAGCCGGGGTG GGTGAAGACTTGCCCTATCAGGAATATTCAGGATCCAGA
